# Weight management and its educational differences among retired individuals living with obesity—a salutogenic approach

**DOI:** 10.1186/s12889-025-23072-w

**Published:** 2025-05-22

**Authors:** Hilla Nordquist, Tea Lallukka, Jatta Valkonen, Anu Joki

**Affiliations:** 1 Department of Healthcare and Emergency Care, South-Eastern Finland University of Applied Sciences, Kotka, 48100 Finland; 2 Department of Public Health, Faculty of Medicine, University of Helsinki, Helsinki, 00014 Finland

**Keywords:** Salutogenesis, Sense of coherence, aging, Obesity, Socioeconomic inequalities, Weight management, Qualitative research

## Abstract

**Introduction:**

This salutogenic-oriented study focuses on weight management among retired individuals living with obesity. We also consider individuals’ educational level, as higher education has been linked to enhanced health and well-being, and improved access to and utilization of various resources. Our research question was: How do retired individuals with low or high levels of education living with obesity implement weight management in their daily lives, from the salutogenic approach?

**Methods:**

Twenty retired individuals with a body mass index (BMI) of at least 30 kg/m^2^ participated in individual qualitative interviews in 2023. The participants were selected from a Helsinki Health Study cohort of retired former employees of the City of Helsinki, Finland. Half of the interviewees had a low level of education, and the other half had a high level of education. Women and men were equally represented. We analyzed the data using a deductive-inductive content analysis.

**Results:**

Four main categories were formed: 1) visionary life management; 2) daily life supporting well-being, 3) enjoying food as an essential part of life; and 4) feeling supported. The main categories had altogether eleven generic categories, and they further had eleven sub-categories. The main categories and most of the generic categories and sub-categories were similar across the low and high levels of education groups, but both groups still had specific characteristics.

**Conclusion:**

A salutogenic orientation, which focuses on health and well-being rather than illness, was evident in many ways in how participants approached their weight management. Educational differences were moderate overall and hinted at specific characteristics of how the resources were utilized. Comprehensibility, manageability, and meaningfulness were evident in the results for both educational groups. According to the results of this study, retired individuals living with obesity implement weight management strategies in their daily lives in ways that are suitable and meaningful for their individual circumstances. They make thoughtful yet enjoyable food choices, select forms of physical activity that motivate them, and spend active quality time with the important people in their lives.

**Supplementary Information:**

The online version contains supplementary material available at 10.1186/s12889-025-23072-w.

## Background

Obesity is a complex, multifaceted public health and societal challenge [1, 2]. Traditionally, research on obesity has been dominated by a pathogenetic perspective, focusing primarily on the risk factors and physiological mechanisms that contribute to the development of obesity and subsequent complications [3–6]. It is often noted that living with obesity presents a particular challenge for older individuals who are at a higher risk of chronic diseases [4–8]. Moreover, transitioning into retirement often changes daily routines, social structures, and access to health services, thus altering the available supportive resources [8–11]. These changes present both challenges and opportunities for implementing health-promoting behaviours [12], see also [8], p.185–187 and [12], p.249–251). Furthermore, weight management at an older age is complex, as dietary choices and physical activity must be specifically tailored to age-related physiological needs to ensure that the benefits outweigh the potential risks [6, 13, 14]. Thus, personalized weight management strategies are essential from both quality of life and public health perspectives for retired individuals [5, 6, 14–18].

The salutogenic model of health, salutogenesis, developed by Aaron Antonovsky (1979) [19, 20] posits that life experiences shape one’s sense of coherence (SOC), meaning one’s orientation towards life to be comprehensible, manageable, and meaningful to varying degrees. SOC is a personal approach to thinking, being, and acting. It is characterized by an inner trust that enables individuals to identify, utilize, and benefit from the resources available to them to effectively handle stressors and manage tension. This process affects one’s position on a continuum between total ill health (dis-ease) and total health (ease). The salutogenic approach is particularly used in health promotion research and practice, as it requires a shift from focusing on the origins of risk factors and diseases (pathogenesis) to the sources of health and assets for positive health [19, 20]. There is evidence that SOC is essential for a healthy aging experience and that SOC can even increase after retirement if an individual’s lifestyle is structured to support it [19, 21]. Further, Seah et al. [22] suggest, following the salutogenic orientation, that by redefining health as the pursuit and achievement of valued accomplishments, optimal health for an individual can be attained at an older age regardless of physical condition.

In this study, we also take into account an individual’s educational background, which is a suitable additional perspective for a salutogenic-oriented study [8]. The salutogenic theory includes generalized resistance resources (GRRs) and specific resistance resources (SRRs) [20, 23, 24], which can be seen as having many direct and indirect connections to educational differences. GRRs are resources that support individuals in handling life’s stressors and promote health and wellness. GRRs can, for example, be physical, psychological, social, or cultural factors that help maintain and improve SOC [23]. While GRRs are broad-based resources useful in various stress situations and generally promote health, SRRs are specific resources related to particular situations that help individuals cope with concrete stressors [24]. Higher educational levels are connected to better health and wellness even among older individuals [25–27] and can generally expand what GRRs and SRRs an individual has access to and how one can utilize them. According to previous evidence, higher level of education broadens access to and utilization of social support [15, 28, 29], which enhances coping with stressful life events (see [12], p.250) and is associated with weight management strategies suitable for the individual circumstances of older people [15, 30, 31]. Additionally, education improves the ability to understand and navigate the healthcare system [32–34], increases access to specialized services [35, 36], and improves digital competencies needed to navigate modern online health services by facilitating efficient management and access to health information [37].

Koelen et al. [8] discussed how professionals typically focus on negatively phrased topics, such as obesity, with an emphasis on the problems and limitations associated with aging. However, older individuals often shift their focus to supportive factors, such as social environments, existing resources, and the ability to make their own decisions [8]. Encouraged by this perspective and utilizing the theory of salutogenesis [8, 12, 19, 20, 23, 24] as well as the insights from previous qualitative salutogenic studies [38, 39], the aim of this study was formulated based on the premise that retired individuals who have lived with obesity for an extended period can offer valuable insights into approaching weight and weight management as a part of their lives and wellness. Our aim was to examine the salutogenic orientation concerning the weight management of retired individuals living with obesity with either a low or high level of education. Our research question was: How do retired individuals with a low or high levels of education living with obesity implement weight management in their daily lives, from the salutogenic approach?

## Methods

In this qualitative, interview-based study, the participants were retired individuals with a body mass index (BMI) of at least 30 kg/m^2^, and with a low or high levels of educational background. We utilized a salutogenic-oriented approach [8, 12, 19, 20, 23, 24, 38] to enhance and deepen the understanding of the participants’ individually described health- and well-being-supporting resources, as well as their contents and meanings in the context of weight management [40]. The Consolidated Criteria for Reporting Qualitative Research (COREQ) checklist was used in the reporting [41].

### Participants

The participants were selected from the Helsinki Health Study (HHS) [42, 43] cohort of retired former employees of the City of Helsinki, Finland, who participated in a health survey in 2022 and expressed their willingness to participate in interview studies. The detailed selection of the participants has been reported elsewhere [44]. This study involved 20 participants, half (*n* = 10) with a low level of education (vocational school, equivalent, or lower) and the other half (*n* = 10) with a high level of education (matriculation or college examination, or higher), reported in a baseline survey in 2000–2002. The low level of education group had a mean age of 67 years (range 61–75 years), and their mean BMI computed from self-reported height and weight was 33 kg/m^2^ in the 2022 survey. Six were women and four were men. The high level of education group had a mean age of 70 years (range 65–77 years), and their mean BMI was 34 kg/m^2^. Five were women and five were men. Of our participants, 15 had retired due to age and 5 due to disability. The sociodemographic, socioeconomic, and health-related characteristics of the participants are summarized in Supplementary Table 1.

### Interviews

Before the interview, the last author (AJ) sent an information letter and a consent form to the participants through email. At the interview site, the participants signed a physical copy of the consent form. They voluntarily agreed to participate in the study and provided their consent for the utilization of their responses to the HHS questionnaire. The interview guide consisted of nine themes (Table 1), including several clarifying additional questions. To develop the interview themes and the semi-structured interview guide, we used the results of weight-loss and weight management studies [45–48], the goals of the broader research project (qualitative research of the HHS [44]), and discussions within the research team. The compatibility between the themes discussed in the interviews and the aim of this study have been considered in more detail in the Strengths and limitations section.

Before starting the interviews, the ethical issues were addressed. The participants were informed about the study’s aim, confidentiality, and anonymity. They were also informed that they could withdraw from the study at any time, without any negative repercussions. The last author (AJ) conducted face-to-face interviews (in Finnish) from May to September 2024, held in locations selected by the participants. The interviews were conducted either in a quiet university office, at the participants’ homes, or in public libraries. The interviews were conducted privately between the interviewer and the participant, with one exception where the participant’s spouse was present. However, we did not include any comments made by the spouse in the analysis. There was no prior relationship between the interviewer and any of the participants. The interviews were on average 72 min long (low level of education group: average 75 min, range 51–101 min; high level of education group: average 68 min, range 50–86 min). The interviews were audio-recorded and transcribed verbatim. The detailed description of the interviews is reported elsewhere [44].

Confidentiality was maintained by assigning identification codes to the participants. The last author (AJ) removed all references that could be used to identify the participant from the data after the transcriptions were completed. The data were stored securely, and access was limited to the members of the research team. In the reporting phase, the findings were presented in a way that ensured the anonymity of the participants.

**Table 1:** The interview guide for the retired participants of the Helsinki health study, interviewed in 2023

Theme	Opening question
1. Life situation	Could you describe your current life situation?
2. Lifestyle habits	What are your thoughts on your lifestyle habits (nutrition, exercise, smoking, alcohol, sleep/sufficient rest)?
3. Food relationship	How would you describe your relationship with food?
4. Eating habits	How would you describe your eating habits?
5. Exercise	How would you describe your relationship with exercise?
6. Thoughts about weight	What are your thoughts on your weight?
7. Weight management experiences	What are your thoughts on weight management, and your experiences with healthcare services concerning weight management?
8. Public discourse	What do you think about the public discussion related to weight and weight management?
9. Vision	If you had an unlimited ability to influence society and the environment, how would you implement weight management?

### Analysis

Before the analysis, the first author (HN) deeply familiarized herself with salutogenesis [8, 12, 19, 20, 23, 24] and its applications in qualitative research [22, 39, 49–51]. This included the study of its operationalization and use in designing the quantitative questionnaire (Salutogenic Health Indicator Scale) [52], its qualitative validation [53], and the qualitative considerations of salutogenesis (particularly SOC) as discussed by Antonovsky et al. [38].

This study utilized a deductive-inductive content analysis [54]. Thus, the aim and research questions were theory-driven, but the responses to the research question were analyzed mainly inductively, taking into account the terminology and connections described in the theoretical framework. Consequently, the reporting of the results does not strictly follow the elements of the salutogenic model of health (Antonovsky 1979; see page 13 in [20]) but is inductively analyzed within the salutogenic framework.

The analysis followed the content analysis process described by Elo et al. [54] and Elo & Kyngäs [55]. The first author (HN) conducted the analysis. The analysis began with careful familiarization with the interview material and its repeated reading. Then the analysis units were defined as words, sentences, and conceptual wholes that answered the research question and had a salutogenic orientation (not pathogenic) were highlighted. These parts of the data were then reduced, meaning their content was condensed without altering the meaning. After this, the reductions, later referred to as codes, were extracted from the original material, and the analysis of the low level of education (total of 206 codes) and high level of education (total of 235 codes) groups continued separately but simultaneously. The participant number (corresponding to the interview order, 1–20) was included in each code, which enabled later quantification [54, 56].

First, the codes collected from the low-education group were preliminarily analyzed by combining similar codes. The same procedure was then applied to the high education group. Then, the preliminary groupings, still containing all the codes, were reviewed together while keeping the interview material from the low and high levels of education groups separated. Similarities and differences were identified within the material, and the scope of the preliminary groupings was examined. The grouping review was an iterative process, at the end of which the sub-categories and general categories were formed and named based on their content [55]. Some content was initially so broad that it ended up in the general category level without sub-categories as the analysis progressed. General categories were then combined into larger main categories based on the content and named accordingly. Content that did not have a counterpart in the other group’s material but still was a clear, distinct content item formed either a sub-category or a general category for that specific education group only. These were marked with colors in the figure that hierarchically presented the results and are also clearly separated in Tables 2, 3, 4 and 5, in which the results are presented.

Finally, the number of participants from each educational group who had brought up the content in each category was counted [54, 56]. The results were reported in tables separately for the low and high levels of education groups following the hierarchical analysis figure. This approach prevented the mixing of the insights and made the specific characteristics of each group visible. Quotations from the original interview material were provided to validate the analysis. In the quotations, “XX” signifies the removal of personal information.

Throughout the analysis process, the content was reviewed to ensure alignment with the salutogenic framework, while the result hierarchy remained inductive. The original material was revisited several times to confirm participants’ original perspectives. The first author (HN), who conducted the analysis, and the last author (AJ), who was the interviewer, discussed the analysis and reviewed the results together. All authors participated in the interpretation of the results. Atlas.ti version 24 was used in the analysis process.

## Results

**Fig. 1:**
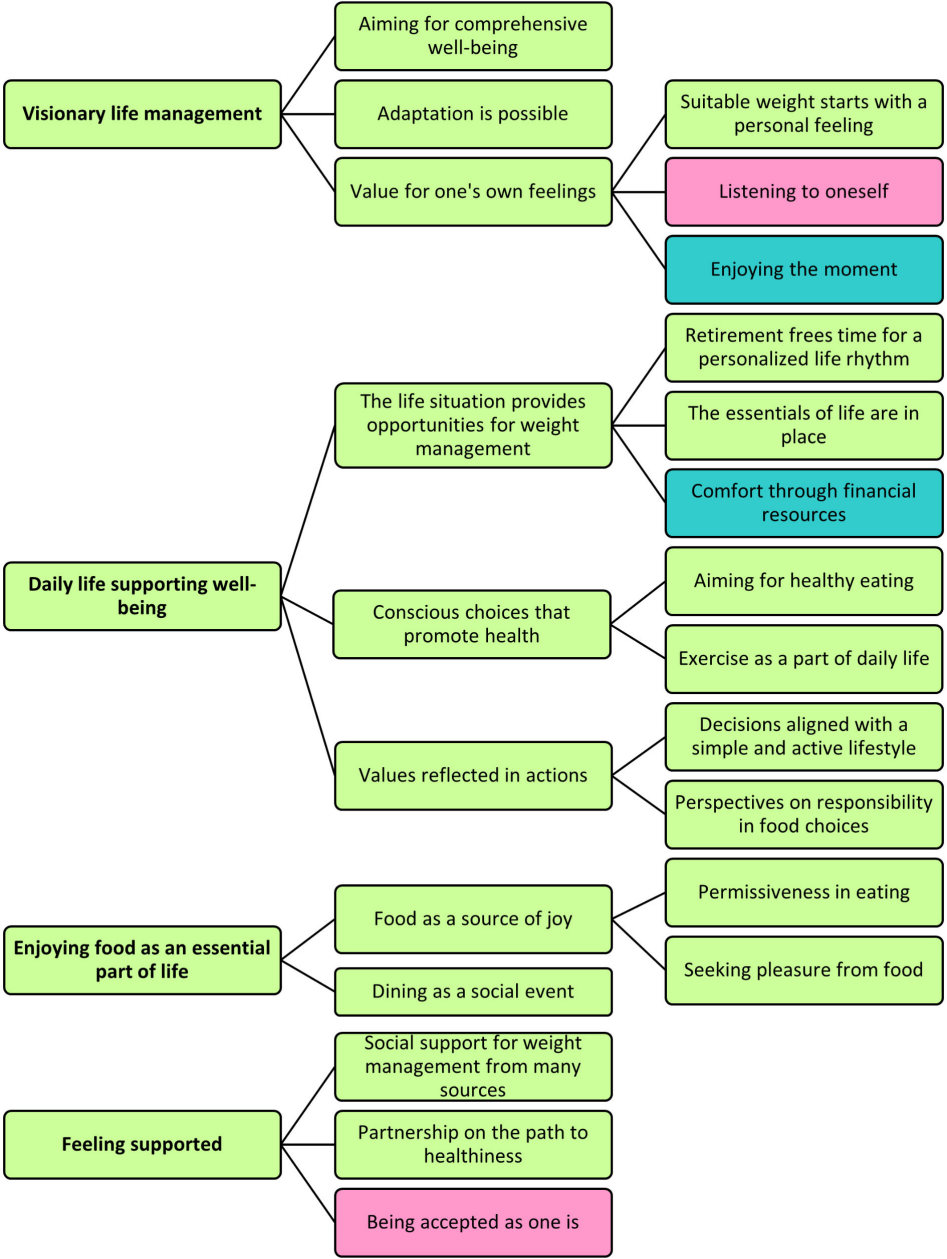
The results are presented as main categories (left), generic categories (in the middle), and their sub-categories (right). Green indicates that the category is common for the low and high levels of education groups. Pink indicates that the category was found only in the low level of education group. Turquoise indicates that the category was found only in the high level of education group

The answers to the research question “How do retired individuals living with obesity and with a low or high levels of education implement weight management in their daily lives, from the salutogenic approach?” formed four main categories, eleven generic categories and eleven sub-categories (Fig. 1). Most of the categories were common across the low and high levels of education groups, but one generic category and one sub-category were found only in the low level of education group, while two sub-categories were found only in the high level of education group.

The results are presented in Tables 2, 3, 4 and 5 by main categories, including the content of each main category. The first main category, *Visionary life management*, is presented in Table 2 and has three generic categories, of which *Aiming for comprehensive well-being* and *Adaptation is possible* were left at the generic category level during the analysis process due to the broadness of the content. The third generic category, *Value for one’s own feelings*, included three sub-categories, one of which appeared only in the interview material from the low level of education group, and one of which appeared only among the high level of education group.

The second main category, *Daily life supporting well-being*, had three generic categories, all of which also had sub-categories identified in the material (Table 3). One of these sub-categories appeared only in the interview material from the high level of education group.

The third main category was *Enjoying food is an essential part of life* (Table 4). This main category had two generic categories, one of which also had sub-categories. All categories were identified in both the low and high levels of education groups.

The fourth main category, *Feeling supported*, had three generic categories, under which there were no sub-categories due to the lack of further details in the content (Table 5). Of these generic categories, *Being accepted as one* appeared only in the interview material from the low level of education group.

**Table 2:** Content of the main category 1: Visionary life management

Generic categories	Generic category content: Low level of education	Generic category content: High level of education	Sub-categories	Sub-category content: Low level of education	Sub-category content: High level of education
**Aiming for comprehensive well-being**	Participants (n = 7) described a positive mindset and self-appreciation as key motivators for weight management and overall well-being. They emphasized maintaining functionality and improving their daily energy levels. Additionally, participants highlighted the importance of recognizing and addressing harmful thought patterns and relationships, the ability to make rational choices instead of emotional ones and prioritizing adequate rest as significant factors in their approach to well-being.	Participants (n = 6) described healthy lifestyle habits as essential for a longer life. An awareness that their choices impact their long-term well-being motivated the participants to make more mindful decisions regarding their diet and physical activity. Maintaining functionality, such as reducing strain on joints and supporting muscle strength, was highlighted as important.	-		
**Adaptation is possible**	Participants (n = 9) described valuing the small things in life and adapting to changing circumstances, such as new financial realities and roles in retirement. A positive attitude and a survivor’s mindset were seen as being helpful in overcoming challenges. Participants emphasized maintaining healthy lifestyle habits while preserving flexibility and avoiding unnecessary stress. They noted the importance of putting things into perspective and focusing on what is going well in their lives. *“In life in general, when something starts to bother you, you should also think about what things in your life are actually going well. Because if you lose some of those, then you really won’t have them anymore. So, you should work on improving the things that aren’t going well, but at the same time, start appreciating and cherishing the things that are going well. That’s how you gain the strength to face difficulties. If something is bothering you today, better times are on their way.”* (Participant 1, low level of education)	Participants (n = 6) described learning to accept the limitations in their lives, such as physical ailments and illnesses, and adapting their daily routines accordingly. These positive attitudes and satisfaction with their current life situation were considered keys to living fully and focusing on what brings joy. Life experience had shown them that medication and physical activity could support adaptation and help manage challenges. The ability to accept oneself as one is was described as bringing a sense of peace.	-		
**Value for one’s own** **feelings**			**Suitable weight starts with a personal feeling**	Participants (n = 6) emphasized that weight loss should stem from their own motivation rather than external pressure or comparisons with others. They highlighted the importance of being at peace with themselves and their weight and overall condition, as well as prioritizing well-being over the aesthetic significance of weight. Recognizing personal responsibility and understanding life’s priorities were described as helping the participants focus on what truly matters for them. An ideal weight was seen as a reflection of self-acceptance rather than a specific number on the scales or appearance.	Participants (n = 6) approached their weight management flexibly, focusing on supporting well-being without causing stress. They expressed satisfaction with their bodies and self-acceptance, valuing functionality and ease in daily activities over external goals. Weight management was described as emphasizing joy and lightness of spirit without strict limitations. The participants noted that their weight did not define their worth or opportunities in life. Acceptance, contentment, and finding a personally suitable balance were seen as key to their approach. *“Yes, body image positivity—it hasn’t always been positive, to be honest. When I was probably, what, in my twenties—25 or 30 years ago at this weight—it initially worried me, frustrated me, and all sorts of things. But when I couldn’t make sense of it or change it myself, I eventually accepted it. And of course, a general sense of body image does have an influence, but it hasn’t affected me in a way that I’d feel the need to force myself into a mold to be a certain way. I’ve walked through the same doors as everyone else with this body.”* (Participant 20, high level of education)
			**Listening to oneself**	Participants (n = 8) valued realistic and personalized approaches to health and well-being. They emphasized a worry-free mindset and understanding what works best for them and setting boundaries according to their needs. A realistic attitude was seen as guiding their life management, with excessive efforts towards weight management perceived as potentially harmful to other areas of life.	-
			**Enjoying the moment**	-	The participants (n = 5) highlighted the importance of enjoying life and living in the moment. Gratitude for their wellness and each new day lived, was described as bringing joy. Letting go of unnecessary worries was seen as supporting a positive outlook on life. *“I’m such an optimist that I try to leave out all the unnecessary worrying. (…) If you are a bit like me, then it’s all good. Let’s enjoy it. You never know what tomorrow will bring.”* (Participant 12, high level of education)

**Table 3:** Content of the main category 2: Daily life supporting well-being

Generic categories	Sub-categories	Sub-category content: Low level of education	Sub-category content: High level of education
**Life situation creates opportunities for** **weight management**	**Retirement freed time for a personalized life rhythm**	Participants (*n* = 4) described retirement as freeing up resources and time for exercise, healthy routines, and shared moments with the family. Life was perceived as more flexible, as being no longer dictated by others’ demands, with self-care taking a center stage in their lives. Retirement was seen as enabling the prioritization of personally meaningful activities.	Participants (*n* = 7) described retirement as bringing freedom and the opportunity to define their own lifestyle and rhythm. They enjoyed not being tied to schedules and appreciated the ability to plan their lives flexibly, avoiding stress and haste. Days were centered around their personal needs, such as getting sufficient sleep, engaging in enjoyable activities, and doing things at a relaxed pace. The participants valued the freedom to focus on pleasant pursuits while leaving behind unnecessary obligations.
	**The essentials in life are in place**	Participants (*n* = 4) described a calm mindset, a healthy sleep routine and abstinence from substances as key factors contributing to their overall well-being every day. Emotional eating was noted to have diminished due to improvements in their life circumstances and environment. Medication was also seen to support their daily weight management.	Participants (*n* = 4) described their lives as balanced, with their illnesses being well-managed and having sufficient resources to support their daily activities. Optimal sleep conditions, a supportive environment, and assistive sleep devices were noted as enabling calm and restful sleep, contributing to their overall daily well-being.
	**Comfort through financial resources**	-	Participants (*n* = 7) described their current financial situation as sufficient, allowing them to make desired purchases without significant limitations. This included high-quality food and wellness-supporting items, such as an electric bicycle. Expensive weight-loss medications were also noted to be within reach for some. *“And then one very important thing is exercise*,* that I can do what I’m able to do based on my illness. And now it’s been wonderful*,* especially this electric bike. When I got it*,* it allowed me to cycle to my daughter’s place… We live in (XX) and my daughter lives in (XX)*,* so I’ve already managed to cycle there twice. It’s given me such a great feeling to be able to move around.”* (Participant 14, high level of education)
**Conscious choices that promote health**	**Aiming for healthy eating**	Participants (*n* = 10) described paying particular attention to healthiness, nutrient intake, and maintaining a regular meal schedule. They described the ability to avoid overeating and adjust food quantities as needed. Eating in moderation was associated with a sense of satisfaction and well-being. *“I do eat vegetables. I make sure to eat the recommended half a kilo a day—or as I like to call it*,* hays. I have a regular grated vegetable portion that I eat every day. I buy it ready-made*,* it’s easy to eat—it’s 250 grams*,* and I eat one portion during the day and another in the evening. And of course*,* I also include vegetables with my meals to make sure I get as much fiber as possible.”* (Participant 9, low level of education)	Participants (*n* = 9) reported following healthy and balanced eating habits, prioritizing a diverse diet that includes vegetables, fish, and wholesome ingredients. A regular meal schedule and moderate portion sizes were emphasized, with efforts to align food choices with nutritional recommendations.
	**Exercise as a part of daily life**	Participants (*n* = 5) reported enjoying a variety of physical activities, taking advantage of senior group offerings, and prioritizing activities they found enjoyable. Functional physical activities, such as everyday exercise, were particularly favored. *“But exercising itself has always been something for me—more or less—but I need to keep moving.”* (Participant 8, low level of education)	Participants (*n* = 9) engaged in diverse and regular physical activities, enjoying both guided and independent exercise. They favored activities that felt good and brought joy, finding ways to stay active while accommodating physical limitations. Exercise was described as a means to relax and enhance their overall quality of life.
**Values reflected in actions**	**Decisions aligned with a simple and active lifestyle**	Participants (*n* = 6) described living in a planned and balanced manner, preparing meals for several days in advance while considering family preferences, and favoring affordable and practical options, such as frozen soups. Physical activity was integrated into daily life through functional exercise, including berry-picking, general summer cottage chores, and yard work. Proximity to recreational facilities and affordable options, such as museums and community tasks, supported an active lifestyle. Weight management was noted to facilitate everyday tasks (for example, tying shoelaces).	Participants (*n* = 6) described living in a structured way, preparing meals in large batches, such as soups, and freezing them to simplify daily life. Walking was often combined with purposeful activities such as errands, and the positive effects of exercise were frequently noted. Summer cottage life and outdoor work were highlighted as supporting weight management. Family dietary preferences, such as a partner’s preference for steak, influenced food choices.
	**Perspectives on responsibility in food choices**	One participant described valuing domestic products, purchasing exclusively Finnish food. They emphasized minimizing food waste by carefully buying and preparing only what would be consumed.	Participants (*n* = 6) reported planning their meals carefully to minimize waste. They prioritized domestic, healthy, and unprocessed ingredients, even when these were more expensive. Meat consumption had decreased in recent years, with vegetables becoming a prominent part of family meals.

**Table 4:** Content of the main category 3: Enjoying food is an essential part of life

Generic categories	Generic category content: Low level of education	Generic category content: High level of education	Sub-categories	Sub-category content: Low level of education	Sub-category content: High level of education
**Food as a source of joy**			**Permissiveness in eating**	Participants (*n* = 4) allowed themselves small treats in daily life and adopted a more relaxed approach on special occasions, such as celebrations. Portion control was used to keep indulgences manageable. This flexible attitude was seen as supporting overall well-being.	Participants (*n* = 8) described following a flexible and permissive food philosophy, incorporating small treats, such as chocolate, into their lives without guilt. Balance was maintained through variety and moderation, and occasional indulgences were not seen to disrupt their weight management.
			**Seeking pleasure from food**	Participants (*n* = 6) viewed food as a source of enjoyment and emphasized both its flavor and visual presentation. They particularly appreciated high-quality, carefully prepared meals and engaged in mindful, slow eating. The participants prioritized foods they enjoyed and balanced indulgence with practices such as taking moments of rest as part of the dining experience. Food was seen as a comprehensive experience.	Participants (*n* = 9) approached eating calmly and mindfully, focusing on savoring flavors and dedicating mealtimes to an undisturbed experience. Food was described as both a hobby and a passion, with participants enjoying diverse cooking and experimenting with new recipes. Eating was seen as both a physical and mental pleasure, incorporating aesthetics such as beautiful table settings and visually appealing dishes. Participants described eating as one of life’s greatest joys. *“I love going out to eat with a friend*,* choosing a great restaurant*,* trying this and that dish*,* and tasting everything. It was especially wonderful for me when we travelled abroad*,* finding out what people eat in different countries. Since I wasn’t interested in drinking*,* it was lovely to go out and enjoy some good food. So*,* food is definitely an important part of life—not that I would say life revolves around it*,* but it’s definitely a huge factor. It’s like the richness of life*,* just like having a good book*,* a comfy chair to sit in*,* or a beautiful park. It’s one of those elements of life.”* (Participant 19, high level of education)
**Dining as a social event**	Participants (*n* = 5) emphasized the social significance of meals and regarded shared family mealtimes as an important part of the day. They organized special moments, such as dining out with a spouse or friends, or participating in communal dining outings. Shared meal experiences were described as valuable and meaningful. *“Our family has these shared mealtimes. So*,* it’s not necessarily that you’re very hungry at that exact moment when we have*,* for example*,* a family dinner. But it has created this rhythm that around this time we have our family dinner*,* and we’re together. It’s a social event.”* (Participant 10, low level of education)	Participants (*n* = 5) described food and mealtimes as highly social events that bring together family, friends, and loved ones. They enjoyed cooking together with others and turned mealtimes into memorable experiences, such as preparing meals for friends and organizing thoughtfully planned dinners. Shared meals with grandchildren and a spouse, as well as long, communal dinners with extended family, were particularly valued. Even grocery shopping, such as visits to a market hall, could be perceived as a social experience. Mealtimes were seen as a central aspect of social life and togetherness, fostering joy and a sense of connection.	-		

**Table 5:** Content of the main category 4: Feeling supported

Generic categories	Generic category content: Low level of education	Generic category content: High level of education
**Social support for weight management from many sources**	Participants (*n* = 7) widely utilized social and community resources to support their well-being and weight management. Family, friends, weight management groups, and peer support networks provided essential encouragement, tips, and support. External factors, such as visits to a doctor and group weigh-ins, further boosted motivation. The involvement of family and close ones, such as encouragement from their children, also inspired the participants to increase their physical activity.	Participants (*n* = 5) extensively utilized social networks and peer support in managing their weight and promoting well-being. Family, friends, and other social groups provided encouragement and inspiration through activities such as swimming together, shared lunch times, and group exercise. The positive, non-judgmental atmosphere of weight management and peer support groups was particularly important, helping to shift mindsets and eating habits. The participants actively engaged in volunteer work, supporting others in lifestyle changes and sharing their own experiences. The physical and social opportunities offered by the city and organizations enriched their daily lives, with enjoyment derived from spending time with grandchildren and participating in shared activities.
**Partnership on the path to healthiness**	Participants (*n* = 5) described their partners to have a significant role in their weight management and well-being. The partners provided both physical and emotional support, participating in shared physical activities and adopting a healthy diet together. The caring and constructive feedback from their partners helped the participants recognize the need for change, and a mutual understanding of eating habits made weight management feel natural and sustainable. *“Then I cautiously said to my partner that I’d really like it if they could come with me. I told them*,* “You don’t have to climb the stairs [referring to specially made stairs to help keep people fit] but just come along with me.” And [the partner] did*,* and that’s how it started for them too. Since then*,* we’ve gone and used all the fitness stairs in our city*,* I think.”* (Participant 5, low level of education)	Participants (*n* = 4) and their partners lead a balanced and active life, combining both individual and shared hobbies and mutual time at home. They engage in arranged physical activities and groups together, while also supporting each other’s separate interests, such as golf.
**Being accepted as one is**	Participants (*n* = 3) valued psychologically safe and accepting relationships where they could be themselves. They received widespread acceptance from their family, just as they are. Among friends, diversity in general was seen as a strength and valued.	-

## Discussion

In this study, our aim was to examine weight management implementation practices in daily life using a salutogenic approach. We focused on retired individuals with high or low levels of education who were living with obesity. The results formed four main categories: (1) visionary life management; (2) daily life supporting well-being; (3) enjoying food as an essential part of life; and (4) feeling supported. In addition, a key finding was that differences between educational groups were moderate. The results were similar across the low and high levels of education groups, but both groups still had specific characteristics.

In our results, there were references to weight management being implemented in ways that are suitable and meaningful for individual circumstances, such as making thoughtful yet enjoyable food choices, selecting forms of physical activity that motivate them, and spending active time with important people in their lives. Although the participants described changes that the transition to retirement had brought to their lives and weight management, in line with previous studies [8–11], the results demonstrate several positive views on opportunities to implement behaviors, enhancing health and well-being in their new life situation [12], see also [8], p.185–187 and [12], p.249–251). For example, the ability to make their own decisions [8, 57] was emphasized in the participants’ responses. It was also linked to the autonomy enabled by retirement, allowing for a self-directed daily routine. The participants reflected on their life circumstances and aligned their personal desires with their responsibilities and family situations.

SOC components were evident in our results in various ways. For example, the participants showed a clear understanding of their well-being and the importance of certain behaviors and structures for weight management, such as healthy eating and exercise. These findings align with the comprehensibility component of SOC [58]. From a salutogenic perspective, understanding the situation enables them to cope more effectively with stress and challenges because what is comprehended is easier to manage. Flexible attitudes to weight management were evident in the results, as participants implemented weight management strategies tailored to their lives and utilized their available resources as support. Limitations, such as physical activity restrictions or occasional indulgence, were not seen as threats to overall well-being, and in general, the results showed the participants were adaptable to different challenges and changes. These findings reflect the manageability component of SOC [58]. The meaningfulness component of SOC [58] is also visible in our results in various ways. For example, the connection of food to both enjoyment and social relationships was clearly part of the meaningfulness in the participants’ lives, which was openly expressed during the interviews. The reflections on what would be a suitable weight also included many descriptions of self-acceptance and prioritizing the feeling of well-being, similar to a qualitative study by Seah et al. [22], which explored perceptions of healthy aging among older adults in an SOC framework. Our results can also be seen as demonstrating the participants’ sense of SOC existence, reflecting their experience and confidence in being able to be themselves.

In this study, the analysis considered the participant’s educational background. While individuals with a higher educational level have better health and well-being [25–27], educational differences were mainly moderate in this study with a salutogenic approach. Notably, the results did not highlight that the high level of education group experienced better abilities or opportunities for weight management compared to the low level of education group. Many of the categories formed through the content analysis were similar for both educational groups. It is important to note that the participants in our study had a BMI of at least 30. Thus, differences might have emerged if there had been variation from low to high BMI.

Nevertheless, there were specific characteristics in the content that justified maintaining the distinction between the groups and highlighting these differences in the results. It can be cautiously assessed that the high level of education group described a clearer connection between their personal choices and future health, and their perspectives on adapting to challenges and changes reflected more of an effort to enjoy life fully. In the low level of education group, there appeared to be more adaptation through acceptance, realism, and striving for balance and good mental health. All these can be seen as GRRs, arising from cultural, social, and environmental living conditions [8, 23, 59], as well as the general experience of SOC—the ability to cope with everyday life—and as a conscious effort towards healthy aging (see [8]), which is defined by WHO as *“the process of developing and maintaining the functional ability that enables well-being in older age”* [60].

Sufficient financial resources were evident in the results only in the high level of education group, for example, through the purchase of desired food, the acquisition of electric bikes to remove barriers to physical activity, and access to expensive weight-loss medications. In the low level of education group, adapting to their new financial realities during retirement was mentioned, but it should be noted outside the analysis that there were no broad descriptions of financial limitations within the low level of education group either. This non-appearance of financial factors in the results may be explained by the fact that all participants had been municipal employees at least at some point during their careers before retirement, and income inequality among workers in Finland is relatively modest at the time of this study [61]. In the salutogenic theory, financial resources are considered part of GRRs [8, 23], but their use in the right contexts may also reflect the ability to utilize SRRs purposefully [8, 24]. For example, the ability to leverage healthcare services and group-based peer support for personal well-being reflects the capacity to harness SRRs [8, 24]. In this study, indications of these skills were found in both groups. It can be cautiously concluded that these were perhaps more apparent in the high level of education group, particularly regarding sleep optimization, medication effectiveness, and the utilization of patient organizations.

Evidence-based choices regarding food and exercise that support health and weight management were evident in both groups. The typical non-material resource brought by education [37] was not strongly apparent in the results of this study. However, the high level of education group did emphasize perspectives related to responsibility more. However, the analysis only considers these perspectives as expressed in terms of resources (adding value to the participants). Thus, both groups may make similar responsible choices. Nevertheless, knowledge and intelligence are factors of GRRs [23].

In Finland, reliable health information is widely available (see [62]), and both groups’ results showed many references to professional and social weight management-related contacts, where information had been shared. A noteworthy finding was that, alongside health awareness, pleasure-seeking support for well-being, especially regarding the content of food and the meanings attached to it, was emphasized. This result was more multidimensional in the high level of education group, although the same core findings were present also among the low level of education group. The importance of food was also linked to social relationships in the results of both groups. Food can be considered a part of culture and a coping mechanism. When viewed this way, the meanings attached to food and eating could perhaps form part of GRRs (see [23], p.93).

Social relationships were seen in the results in various ways, including references to adapting the family situation to one’s daily rhythm, descriptions of diverse social activity, and social support. The results suggest some differences in the descriptions between the low level of education and high level of education groups. According to previous evidence, access to and utilization of social support is higher among those with a higher level of education [15, 28, 29]. Our results showed, though cautiously assessed, that the high level of education group seemed to have a slightly broader social environment. However, the amount of social support cannot be conclusively inferred from our findings. The differences were more related to how social life and support were described. For example, weight management-related relationship descriptions in the high level of education group were more focused on shared activities, while in the low level of education group, descriptions centered more on being present and supportive in daily life, like a tight team.

Only participants from the low level of education group highlighted the experience of social acceptance as part of their authentic self. This might have occurred coincidentally. Both groups, however, referred to self-acceptance. The salutogenic literature describes the feeling of belonging and social inclusion as important in older age groups [8] and recognizes self-esteem [24] and ego identity [23] as part of the GRRs. Overall, social relationships are a significant part of GRRs [8, 23, 46], and the concept of community could be seen as highlighted in a salutogenic orientation [see 8]. In these aspects, our results are well aligned with the salutogenic literature.

Overall, our results suggest that the participants perceived weight management differently, unique to their own lives and circumstances. Considering the qualitative research design, it cannot be ruled out that the researchers have interpreted different meanings from the interviews than the participants themselves attribute to weight management in their real lives. However, the core message is quite clear: weight management strategies should be suitable for individual circumstances, noted also elsewhere (see [63, 64]). The salutogenic literature recognizes that SOC can increase even at a later age [8, 21]. A previous study has concluded that SOC in healthy aging can be enhanced by reducing unpredictability (comprehensibility), promoting actions for physical, mental, and social health (manageability), and fostering reflection to find purpose and meaning in old age (meaningfulness) [22]. Healthy aging involves developing and maintaining functional ability [60], which our participants highlighted, and is also supported by weight management [5, 15–18]. Based on our findings, retired individuals living with obesity would particularly benefit from health promotion efforts that take their social environment into account and boost social activity. Since educational differences are known to be significant in health and well-being [25–27], educational background should also be considered when targeting health-promoting activities. While knowledge on healthy lifestyles exists, utilizing existing resources in daily life and considering individual circumstances could always be further supported.

### Strengths and limitations

This research can be described as a preliminary investigation that provides a foundation for further research on this topic, as the study design has many limitations, but also strengths. This qualitative interview study had participants from the HHS cohort. The authors had access to their detailed background information, which enabled the recruitment of the most appropriate participants (retired, BMI of at least 30 kg/m², and a low or high levels of educational background) for our broader research project (qualitative research of HHS [44]). The study produced, as desired for qualitative research, in-depth information within this target group. We did not aim for broader generalizability, which is not a goal of qualitative research in general [65]. 

One aspect to consider is that the interview framework was originally not tailored specifically to explore the salutogenic model of health, and consequently, did not include questions aligned with this framework. However, once the interviews were finished, it became evident that the interview material could also be analyzed with a salutogenic approach, revealing numerous descriptions of SOC, health-supporting resources, and their significance. Thus, in this study, the participants’ salutogenic orientation was found without being the central focus of the interviews, which can also be seen as a strength for the credibility of the research and the authenticity of salutogenesis as a phenomenon in their lives. Similar post-data gathering observations are described in the study by Husby et al. [50] and further discussed by Antonovsky et al. [38]. Still, acknowledging the complexity of the salutogenic health theory, it is important to note, regarding this study’s confirmability [66], that it is possible that the results of this study include too much interpretation, and interview material specifically focused on salutogenic orientation could result in more precise insights.

Another consideration for the study’s credibility [67] is that we focused on the context of weight management and not on “global orientation”, meaning that neither the interviews nor the analysis explored the interviewees’ fundamental attitude or orientation that guides their way of experiencing and responding to various life situations and challenges (see the three dimension of SOC [8]). Yet, Antonovsky et al. [38] explain that in qualitative interviews, it might be difficult for participants to base their responses on a global orientation to life, as they respond within a specific context. However, examining the global orientation would require data collection about several areas in a person’s life; therefore, a single interview session is immediately a limitation for the credibility [38]. Additionally, the interviews of this study primarily focused on current life situations, but the participants were also asked to describe their weight history and experiences throughout their entire life course. The content relevant to the research question was considered regardless of its temporal dimension.

While this study was initiated with a theory-driven approach (deductive reasoning), the analysis was primarily conducted using inductive reasoning to derive conclusions directly from the data, while still considering the predefined theoretical framework of salutogenesis. The deductive-inductive content analysis is described in a recent methodological article [54], and it can be assessed as well-suited as a starting point, where the interview framework did not include questions aligned with the salutogenic framework. The content analysis approach allowed the quantification of the results [54, 56, 67], which was valued because the aim included the desire to study low and high levels of education groups separately. This aim was maintained, even though the results were largely consistent. The identified differences are important considerations that can serve as a basis for further studies, such as examining the role of educational level or, more broadly, socioeconomic position in the manifestation of a salutogenic orientation or available resources during a life course. The separate reporting of the results also strengthens the transferability of the findings, as the target group is more precisely defined. Moreover, the dependability and confirmability of the study are strengthened by the careful reporting of the research process and the transparent presentation of its limitations [66, 67].

The multidisciplinary background of the research team can be seen as a strength of this study. The first author (HN) is an experienced senior researcher with a broad understanding of qualitative methodology and an educational background in prevention, pedagogy, occupational health, and public health. She is also a licensed healthcare professional with experience in health promotion. The second author (TL) is a professor and the principal investigator of the HHS project, with a background in nutrition sciences and social epidemiology. She is one of the leading researchers in socioeconomic health differences and has focused on topics such as obesity, health behaviors, and health inequalities. The third author (JV) is a postdoctoral researcher with an educational background in health management science and nutrition sciences. She has particularly focused on socioeconomic differences in lifestyle habits and developmental patterns in body weight. The last author (AJ) is a postdoctoral researcher with an educational background in nutrition sciences. She is an experienced qualitative researcher who has predominantly focused on weight management and lifestyle changes. She also has experience in health promotion as a weight management group facilitator. The multidisciplinary background and the collaboration of the research team enabled not only the precise methodology but also deep perspectives when interpreting the results and contemplating their meanings. The mutual discussion of the results between the first author, who conducted the analysis, and the last author, who collected the interview data, strengthens the confirmability of the results [66]. The comprehensive health promotion competence of the researchers also strengthened the utilization of a salutogenic orientation and supported the confirmability of the results.

## Conclusions

This qualitative interview study demonstrated that a salutogenic orientation exists in various ways in the descriptions and meanings that retired Finnish individuals living with obesity expressed regarding their weight management, mainly irrespective of their educational level. For example, free time due to retirement, conscious healthy daily choices, but still with moments of pure enjoyment, and active social relationships supported their well-being and weight management. The SOC components of comprehensibility, manageability, and meaningfulness were evident in the results. Our participants demonstrated a clear understanding of their well-being and showed a flexible approach to weight management, adapting strategies to fit their lives and utilizing available resources for support. The results showed their adaptability to change and face challenges. Food was described as both enjoyment and as a part of social relationships, contributing to the sense of meaningfulness in the participants’ lives. Overall, our results highlight the importance of developing public health policies and health promotion interventions targeted at retired individuals living with obesity, which support flexible everyday health practices and social connections, and consider diverse life situations. In our study, descriptions of resources aligned with a salutogenic orientation were quite similar among retired individuals living with obesity and with either low or high levels of education. Still, based on extensive previous knowledge regarding educational differences in health and well-being, health policies and promotion should consider the educational background.

## Electronic supplementary material

Below is the link to the electronic supplementary material.

Supplementary Material 1

## Data Availability

The datasets generated and analyzed during the current study are not publicly available because they contain participant-identifying information and are sensitive. The participants were assured that the data would be stored only on the University of Helsinki’s secure server. Only members of the group who have signed a strict confidentiality document have access to the data. Please contact Professor Tea Lallukka (tea.lallukka@helsinki.fi) for further details on obtaining access to the interview data analyzed in the study.

## Abbreviations

BMI: Body mass index
COREQ: Consolidated criteria for reporting qualitative research
GRRs: Generalized resistance resources
HHS: Helsinki health study
SOC: Sense of coherence
SRRs: Specific resistance resources
TENK: The Finnish national board on research integrity
