# Supplementary material for: Weight management and its educational differences among retired individuals living with obesity—a salutogenic approach
Source: BMC Public Health. 2025 May 22;25:1892. doi: 10.1186/s12889-025-23072-w (PMC12096649; doi:10.1186/s12889-025-23072-w)
Supplement: Supplementary file 1 — Supplementary Material 1 [file 12889_2025_23072_MOESM1_ESM.docx]

**Supplementary materials**

Title: Weight management and its educational differences among retired individuals living with obesity—A salutogenic approach

Authors: Hilla Nordquist, Tea Lallukka, Jatta Valkonen, Anu Joki

Supplementary Table 1. Characteristics of the retired participants of the qualitative Helsinki Health Study, interviewed in 2023 (n=20).

| **Characteristic** | **N** |
| --- | --- |
| ***Age (mean)*** | 68.6 |
| ***Gender*** |  |
| Woman | 11 |
| Man | 9 |
| ***Type of retirement*** |  |
| Retired due to disability | 5 |
| Retired due to age | 15 |
| ***Marital status*** |  |
| Separated, divorced, or widowed | 8 |
| Married or in a registered partnership | 12 |
| ***Education level*** |  |
| Low (i.e., vocational school, equivalent, or lower) | 10 |
| High (i.e., matriculation or college examination, or higher) | 10 |
| ***Body mass index, kg/m^2^ (mean) ^a^*** | 33.3 |
| ***Self-rated health ^b^*** |  |
| Fair or good | 8 |
| Very good or excellent | 11 |

^a^ BMI = body mass index
^b^ Self-rated health was assessed with the following question and response alternatives: “In general, would you say your health is: excellent, very good, good, fair, poor?”. There were no participants with ‘poor’ self-rated health.
